# Piezoelectric smart biomaterials for bone and cartilage tissue engineering

**DOI:** 10.1186/s41232-018-0059-8

**Published:** 2018-02-27

**Authors:** Jaicy Jacob, Namdev More, Kiran Kalia, Govinda Kapusetti

**Affiliations:** Department of Medical Devices, National Institute of Pharmaceutical Education and Research, Ahmedabad, 380054 India

**Keywords:** Piezoelectricity, Piezoelectric materials, Bone, Cartilage, Tissue regeneration, Electroactive scaffolds, Mechanical stimulation

## Abstract

Tissues like bone and cartilage are remodeled dynamically for their functional requirements by signaling pathways. The signals are controlled by the cells and extracellular matrix and transmitted through an electrical and chemical synapse. Scaffold-based tissue engineering therapies largely disturb the natural signaling pathways, due to their rigidity towards signal conduction, despite their therapeutic advantages. Thus, there is a high need of smart biomaterials, which can conveniently generate and transfer the bioelectric signals analogous to native tissues for appropriate physiological functions. Piezoelectric materials can generate electrical signals in response to the applied stress. Furthermore, they can stimulate the signaling pathways and thereby enhance the tissue regeneration at the impaired site. The piezoelectric scaffolds can act as sensitive mechanoelectrical transduction systems. Hence, it is applicable to the regions, where mechanical loads are predominant. The present review is mainly concentrated on the mechanism related to the electrical stimulation in a biological system and the different piezoelectric materials suitable for bone and cartilage tissue engineering.

## Background

Smart materials are in general discussed to the materials, which can reversibly modify one or more of its functional or structural properties, according to the imposed external stimulus or to the modifications in their surrounding conditions [1]. The external stimulus includes physical (temperature, light, electric or magnetic fields), chemical (pH) and mechanical stimuli (stress and strain). Piezoelectric materials are considered as smart materials owing to the fact that these materials can transduce the mechanical pressure acting on it to the electrical signals (called direct piezoelectric effect) and electrical signals to mechanical signals (called converse piezoelectric effect) [2]. The basic requirement of material to exhibit piezoelectricity depends on its crystal lattice structure and the lack of a center of symmetry [3]. Pierre Curie and Jacques Curie in 1880 have discovered the phenomenon. The word “*piezo*” originates from the Greek word “*piezein”* meaning pressure [4].

Piezoelectric materials have a wide variety of electronic applications such as transducers, actuators and sensors. Moreover, piezoelectric materials have significant applications in tissue engineering as an electroactive scaffold for tissue repair and regeneration. They can deliver variable electrical stimulus without an external power source [5]. The electrical stimulation resulting from piezoelectric scaffold can regenerate and repair the tissues by definite pathways [6]. The piezoelectric scaffolds with optimized properties can produce suitable bioelectrical signals, similar to the natural extracellular matrix (ECM), which has observed during remodeling phenomenon in bone and cartilage [7].

The electro-active scaffolds are most significant in tissue engineering where the electrical stimulation is relevant for the tissue repair or regeneration, such as, neuronal tissue repair, bone and cartilage repair and regeneration etc. [8]. Tissues like bone, cartilage, dentin, tendon and keratin can demonstrate direct piezoelectricity [9]. Collagen is a fiber-like structure and it is major constituent in bone and cartilage, responsible for the piezoelectric property [10]. Due to the piezoelectric property of collagen, it can generate electric signals in response to internal forces. These signals transmitted through ECM to the voltage-gated channels in the cell membrane. Mainly, the osteocyte cells are involved in mechanotransduction and they communicate with other cells such as osteoblasts and osteoclasts. The activation of these channels transmits the intracellular signals to the nucleus, leads to the activation of signaling cascades, responsible for the cellular events such as matrix production, cell growth and tissue repair [11]. Hence, the electro-active scaffolds, which mimics the piezoelectric coefficients of natural tissues may be a suitable approach for the repair and regeneration of skeletal tissues like bone and cartilage.

**Fig. 1:**
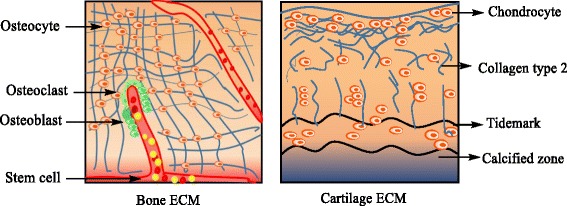
Illustration shows the highly vascularized ECM of bone (Bone ECM) and avascular ECM of cartilage (cartilage ECM)

Bone and cartilage are dense connective tissues, which consist mainly cells and extracellular matrix (ECM) (Fig. 1). In general, ECM consists two main cell types immature and mature, the immature cell in cartilage and bone are chondroblast and osteoblast, respectively. Vitally, the blast cells have the capacity to cell division and further it secretes the ECM. Subsequently the blast cells differentiate into mature cells like chondrocytes and osteocytes, in cartilage and bone respectively. Matured cells are mostly encompassing in conserving the matrix and it has limited capacity for cell division and matrix production [12].

Other cells present in the matrix are fibroblasts, macrophages, adipocytes and mast cells.

ECM is well distributed among the cells, and provides a microenvironment to perform their regular activities and functions. Besides, via ECM, the signals are transmitted to the cell membrane receptors, which activates intracellular signaling cascades and this provides stimuli to the nucleus [13]. The stimuli will further regulate the transcription of several proteins, which have a significant role to regulate cell functionality. Beyond these, ECM can regulate their size and shape according to the changes in the external loads [14]. The characteristic of ECM differs based on the embedded cell type. The bone has rigid/inflexible ECM, while; cartilage has flexible ECM due to the presence of different cells. Further, structurally cartilage is avascular, but all connective tissues including bone are highly vascular.

Generally, ECM structure comprises a hydrated network of glycosaminoglycan chains, with various interwoven protein fibrils and fibers. The bone has abundant ECM, composed of 25% water, 25% organic collagen fibers and 50% crystallized mineral salts [15]. The inorganic mineral salts in the form of microcrystalline, such as hydroxyapatite [Ca_10_ (PO4)_6_ (OH)_2_] confer the hardness and mostly rigidity of the bone [16]. The mineral salts like calcium hydroxide and calcium phosphate combined to form centrosymmetric hydroxyapatite nanocrystals, which further combines with other mineral salts and ions such as magnesium, fluoride and manganese. These crystals were deposited in the network of collagen fibers, which further undergoing a process called calcination. The entire process is initiated by bone formation cells (osteoblasts) [15]. Compact bone has mostly type I collagen and has the piezoelectric coefficient approximately 0.7pC/N [17].

The ECM of cartilage comprises of collagen (type II, VI, IX, X and XI), proteoglycan, non-collagenous proteins and tissue fluid. Collagen is strong and flexible structure and can resist the pulling forces [18]. Among all, cartilage is compose of 90–95% type II collagen and the primary function is to resist tension [19]. The piezoelectric collagen can influence the cell membrane receptors and ultimately the nucleus owing to electrical charge alterations in response to functional loads [20].

The deformities and injuries in hard tissues like, bone and cartilage can occur, primarily, due to mechanical trauma and various disease conditions. The osteoporosis, paget disease, ricket, osteomalacia, osteoarthritis, osteomyelitis, and osteosarcoma mainly contribute bone degeneration [21]. Cartilage degeneration is primarily, due to gaut, osteoarthritis, acromegaly and alkeptonuric ochronosis [22].

Conventional therapies include pharmacological treatments such as, estrogens and selective estrogen receptor modulator (e.g. tamoixifen, ralaoxifen and nafoxidine) [23], biphosphonates (e.g. alendronate, zoledronate and pomidronate) [24], anti-inflammatory molcules (e.g. NSAID, indomethacine and aspirin) [25]. However, major limitation of the pharmacological therapies are disease dependent, less effective in case of critical degeneration, lack of site specificity and drug associated toxicity [26]. Surgical intervention is an important treatment choice; the major practices are autograft, allograft, xenograft, bone marrow stimulation, mosacoplsty and autologous chondrocyte implantation. The surgical practices have success rate up to some extent, the major limitations come from; donor site morbidity, due to secondary surgery associated with autograft and allograft. Furthermore, immunogenic rejection and disease transmission as consequence of allograft and xenograft practices [27–29]. Bone marrow stimulation has poor regenerative capacity and the regenerated cartilage has low biomechanical integrity [30], the donor site morbidity also associated with mosiacplasty [31]. The autologous chondrocyte implantation is a costly practice and complicated process, it is not recommended for osteoarthritis [32].

In recent years, researchers are seen tissue engineering approach as an effective alternative for hard tissue regeneration and repair. The advanced tissue engineering methodology is a mutli-displinary technique. It is an amalgamation of engineering and the life science principle for the repair, replace, maintain, or enhance the function of a tissue and related organ. The tissue engineering aspects broadly covers the cell seeded, growth factor implanted, drug loaded and other bioactive molecule loaded scaffolds [33]. Basically, the cell based therapy utilizes various cell types like, mesenchymal stem cells (MSCs), embryonic stem cells (ESCs) and induced pluripotent stem cells (iPSC) [34, 35]. The growth factor such as transforming growth factor-β, bone morphogenic protein − 2, bone morphogenic protein-4 etc. are frequently used in bone and cartilage tissue engineering [36]. Various causes restrict the use of cell-based and growth factor based therapies for cartilage and bone regeneration exercises. In cell-based therapies; chondrogenic and osteogenic potentials differ from their source, cell senescence, unpredictable differentiation because of improper microenvironment, the initial insufficient nutrient and hypoxic condition at implanted site lead to irregular outcomes. The growth factor based therapies are highly expensive, involves complicate experimental process, high instability, hazy selection (no standard criteria for the selection of growth factor), dose related complications, short half-life and scalability [37–43]. Hence, there is a high need of safe and effective alternatives to regeneration and repair of complex tissue like bone and cartilage.

The advancement in material science and engineering to develop specialized materials to crack the baffling problems by introducing so-called smart materials in various applications [44–46]. The smart material is described as, variation of at least one property of material is stable, reproducible and significant, when material is subjected to external stimuli. It is well reported that, the classification of smart materials typically depend on its output response, which includes piezoelectric materials, materials develop stable and reproducible electric signals, when mechanical stresses applied and vice-versa; large deformations can be induced and recovered in presence of temperature or stress variations in shape memory smart materials; temperature responsive materials, pH sensitive materials, self-healing materials and thermoelectric materials etc. [47–49].

**Fig. 2:**
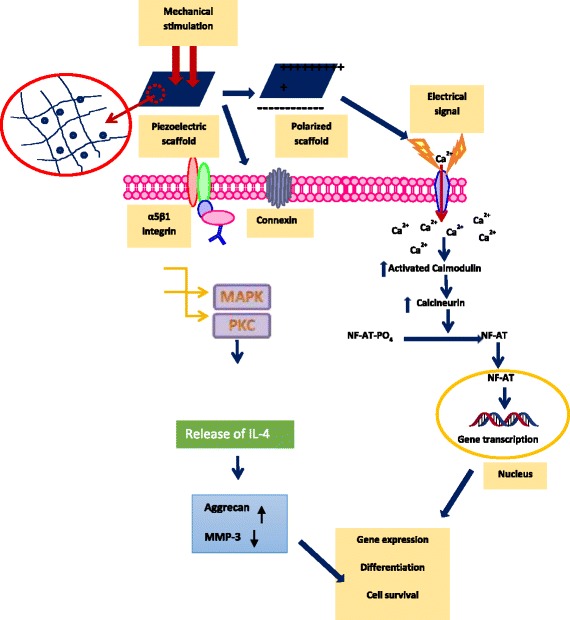
Schematic diagram of ca^2+^ signal transduction pathway and other miscellaneous pathways activate in response to the electrical and mechanical stimulations. The mechanical stimulation on piezoelectric scaffold will result in the electrical signal generation and which will stimulate the voltage-gated ca^2+^ channel. Further increase in the intracellular Ca^2+^ concentration activates the calmodulin (an abbreviation of the calcium-modulated protein) and which will further activate the calcineurin (calcium and calmodulin-dependent serine/threonine protein phosphatase). The activated calcineurin dephosphorylates the NF-AT and it will translocate to the nucleus, where it acts in conjunction with other associated proteins as transcription factors. Also the mechanical stimulation itself can activate the mechanoreceptors present in the membrane and which will lead to the activation of PKC and MAPK signaling cascades. These cascades will result in the synthesis of proteoglycan and inhibition of IL-1, responsible for the breakdown of proteoglycan

Piezoelectricity has shown its strong effectiveness in natural pathways, specifically at the site where the collagen implicated activities. The compressive force on collagen triggers the re-organization of dipole moment and generates negative charges on the surface [50]. The generated charge prompts the electrical stimulation to the cells, leads to the opening of voltage-gated calcium channels. The increased activity of intracellular calcium concentration activates the calmodulin, which subsequently stimulates the activation of calcineurin. The calcineurin dephosphorylates NF-AT (Nuclear Factor of Activated Cells), which further translocate into the nucleus, where it binds co-operatively with other transcription factors to regulatory regions of the inducible genes. These genes further induce the translation of several growth factors like Transforming Growth Factorβ (TGF β), Bone Morphogenetic Protein (BMP) etc. which are responsible for the regulation of ECM production as well as up/ down regulation of several proteins and cellular metabolism [38, 51]. Various studies were reported that the electrical stimulation can produce TGF β through calcium/ calmodulin pathway and the TGF β is the potential key factor to promote the cellular processes including cell growth and differentiation, extracellular matrix synthesis, inflammation and tissue repair (Fig. 2). These pleiotropic actions of TGFβ are due to its involvement in either inhibition or stimulation of some common regulatory pathways responsible for the cellular events [52]. It is an important growth factor for the formation of bone and cartilage [53].

**Scheme 1:**
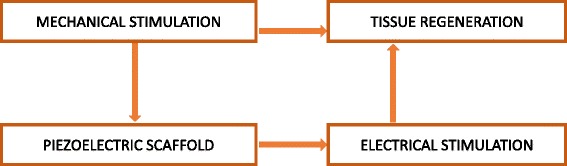
Representing the tissue regeneration in response to the mechanical stimulation on the piezoelectric scaffold. The mechanical force on the piezoelectric scaffold generates the electrical stimulus for enhanced tissue regeneration. At the same time, applied mechanical stress can simultaneously augments the tissue regeneration in predefined signaling pathways

In general, tensile/compression forces acting on the piezoelectric scaffolds generates the electrical stimulation and transfers it to the surrounding cells, promotes the cell signaling pathways, responsible for the growth factor synthesis (Scheme 1). The mechanism behind the conversion of mechanical stimuli into biochemical signals remains elusive [54]. It is well evident that the piezoelectric collagen stimulates the cell proliferation (tissue regeneration) by electrical stimuli via mechanotrasduction. The collagen possesses polar hexagonal crystalline unit and it is primarily responsible for piezoelectricity [55]. The literature strongly suggests that the collagen rich bone converts the functional stresses into electrical stimulations for regeneration and remodeling. The electrical stimulation is largely contributes in cell phynotypic change [56]. Mechanical stimulation has some major constrains like, sensitization of bone cell, age related issue like higher the age poor the regenerative capacity [57]. The mechanotransduction pathway involves the stretch-activated ion channels, α5β1 and an autocrine/ paracrine interleukin-4 (IL-4) loop (Fig. 2) [58]. The α5β1 integrin is a major mechanoreceptor present in the chondrocyte and bone cells [59]. The activation of α5β1 integrin as a result of the mechanical stresses, followed by translocation of the protein kinase C (PKC) to the cell membrane. Hence, the integrin-dependent PKC associated signaling cascades including Ras/Rac dependent MAP Kinase pathway has been activated [60]. The activation of PKC increases the activation of proteoglycan synthesis, inhibits the interleukin-1 (IL-1) induced proteoglycan breakdown and inhibition of proteoglycan synthesis [58].

### Piezoelectric materials

To induce piezoelectric property in the scaffold, the best possible way is to select appropriate piezoelectric material; either, piezoelectric polymer or ceramic or polymer-ceramic composite for fabrication of bio-scaffold. Hence, piezoelectric materials are best suits for biomedical applications, where the electromechanical transduction involves. The property possesses to the material due to lack of center of symmetry [61]. The deformation of such materials results in the development of charges of opposite polarity on opposite faces of crystals. Fundamentally this is due to the separation of the center of neutrality of charges on the crystal lattice as the material is deformed along certain axes. The term applies to some polycrystalline, inorganic materials and some inorganic substances [9]. Piezoelectric materials can also be classifies as piezoelectric polymers and piezoelectric ceramics. The piezoelectric ceramics are included in the polycrystalline class [62]. Piezoelectric materials are using either alone or as a composite in tissue engineering.

### Piezoelectric polymers

The properties of piezoelectric polymers are different from inorganic crystals, since these possess the advantage of processing flexibility. Mechanically, polymers have high strength and high impact resistance as compared to inorganic materials. Structural requirements of piezoelectric polymers are (1) the presence of permanent molecular dipoles (2) the ability to align or orient the molecular dipoles (3) the ability to sustain the alignment once it is achieve and (4) the ability of the material to undergo large strains when mechanically stressed [63]. The piezoelectric polymers, which are used in tissue engineering for cartilage and bone as follows:

#### PVDF (poly(vinylidene fluoride))

PVDF is a best known piezoelectric copolymer with the piezoelectric coefficient of 20 pC/N [64]. Due to its high flexibility and non-toxicity, PVDF have been used for a variety of biomedical applications, from tissue engineering scaffolds to implantable self-powered devices [65]. PVDF-TrFE and barium titanate piezoelectric composite membrane has been reported as charge generator to promote the bone regeneration [66]. Martins et al. were well demonstrated the potential application of PVDF scaffolds in skeletal muscle regeneration. After corona poling of the PVDF scaffolds the formed negatively charged surfaces promote better cell adhesion and proliferation of myoblast cells [67]. The piezoelectric PVDF scaffold has been largely promoting the osteogenic differentiation of human adipose-derived stem cells [68]. A novel piezoelectric actuator device based on PVDF has been demonstrated to effectively stimulate the bone growth at the bone-implant interface by the use of converse piezoelectric effect [69]. Furthermore, PVDF and PVDF-TrFE has been reported for neural tissue regeneration [70]. The PVDF is well known biocompatible thermoplastic polymer. It has high chemical and physical resistance. Still when it expose to the extreme alkaline condition it tends to degrade but not suitable for biological environment [71]. However, the PVDF is a non-biodegradable polymer which limits its applicability in tissue engineering [72].

#### P(VDF-TrFE)

It is a copolymer of vinylidene fluoride (VDF) and trifluoroethylene (TrFE). The copolymer has been demonstrated highest piezoelectric coefficient (30 pC/N) among the polymers [73]. It was reported that the copolymer is cytocompatible and shown positive influence on cell adhesion and cell proliferation [74]. The polymer has an ability to regenerate the different type of tissues like, bone, skin, cartilage and tendon [75].The electrospun nanofibrous based scaffold of PVDF-TrFE copolymer have been regenerated neural and articular cartilage very efficiently [5]. The piezoelectric fibers can be stimulated the differentiable cells into mature phenotype and have an ability to promote stem cell-induced tissue repair [7]. Currently, the blends of polymers for bone and cartilage tissue engineering are gaining more importance. Furthermore, the PVDF and PVDF-TrFE have been blended with starch or cellulose-like natural polymer to develop suitable scaffold structures for tissue repair and regeneration, particularly for bone tissue engineering. The starch or cellulose is blended to produce a porous structure to support tissue growth [76].

#### PHBV (poly- 3- hydroxybutyrate-3-hydroxy valerate)

PHBV is a member of PHAs and it is gaining importance in biomedical field because of its biocompatibility, biodegradability and its thermoplasticity [77]. Moreover, it has longer degradation time than other biocompatible polymer and remarkably it has piezoelectric coefficient (1.3 pC/N) similar to human bone [78, 79]. The studies had been reported the collagen-PHBV matrices for cartilage tissue engineering because of its biocompatibility and more extended biodegradation rate [80]. PHBV has been degraded by enzymatic degradation mechanism, subsequent hydrolysis and release the carbon dioxide [81]. Biodegradable PHBV-HA composite had been demonstrated for bone tissue engineering [77]. Similarly various studies had been reported the use of PHBV as matrices for cartilage and bone regeneration while, the utilization of piezoelectric property of PHBV for bone and cartilage regeneration is not till reported.

#### Polyamides

Polyamides and polypeptides possess piezoelectricity by odd numbered Nylons and peptide (˗CONH) bonds, respectively [64]. Odd nylons (nylons-5, nylons-7) contains even numbered methylene groups and one amide group on each monomer unit. Due to the presence of one amide group, odd numbered nylon results net dipole moment (3.7 D) [82]. The piezoelectric polarization proceeds as a consequence of the stress-induced internal rotation of the peptide bonds [83]. The piezoelectric coefficient (d_31_) for nylon is 3 pC/N at 25 °C and 14 pC/N at 107 °C [82]. Wang et al., have been reported that polyamide-hydroxyapatite composite promotes the osteogenesis by 12 weeks of implantation [84]. Also studies had been reported that the polyamides are applicable for cartilage repair or regeneration as a polymeric matrix. But proper modifications are required to promote the cell attachment and proliferation of chondrocytes [85]. Lack of degradation pattern of the polyamides has limited applications in tissue engineering.

#### Poly-l-lactic acid (PLLA)

Poly-L-lactic acid is a biodegradable and biocompatible polymer, along with it has a large shear piezoelectric coefficient. The piezoelectric shear coefficient of PLLA (d_14_) is − 10 pC/N [73]. Due to its helical structure, it doesn’t require poling for generation of piezoelectricity. Moreover, the mechanical orientation of molecules in the crystals and the quasi-crystalline region is enough to generate piezoelectricity. Fukada, et al. has demonstrated that implantation of PLLA can promote the bone growth in the response of its piezoelectric polarization [86]. PLLA has huge clinical application in orthopedics such as screws and pins, due to its strong mechanical properties. The PLLA is degraded by hydrolytic degradation and the byproduct is PLA, which is nontoxic and water-soluble. The degradable PLLA has been well documented for rapid bone regeneration by consuming its piezoelectric property [73].

#### Biopolymers

Natural polymers are gaining more importance in tissue engineering because of their degradability and low toxicity. More than that, the polymers have offer biological signaling, cell adhesion, cell responsive degradation and remodeling [17]. Meanwhile, their use as a unique scaffold material has often compromise owing to their inadequate physical properties, together with the possible loss of biological properties during formulations. Moreover, appropriate screening and processing are required to avoid the disease transmission and immune rejection. While suitable chemical or physical processing will help to overcome these issues [87].

#### Cellulose

Cellulose is the most abundant natural polymer on earth and it has a piezoelectric property with a shear piezoelectric coefficient (d_14_) 0.2 pC/N [88]. It has large number biomedical applications, due to excellent biocompatibility, high tensile strength and impersonates with biological environment, despite its water content and nanofibrous structure. While, it has a small pore size or the dense mesh formation of fibers limits the cell infiltration. However this can be overcome by the incorporation of proper porogens. Moreover, studies have been demonstrated that the ability of cellulose to promote cellular adhesion particularly chondrocytes, osteocytes, endothelial cells and smooth muscle cells [89]. Hence, it is appropriate piezoelectric material for both bone and cartilage tissue engineering.

#### Collagen

Collagen is a biological protein and vital component of the ECM like bone, cartilage, tendon, teeth and blood vessels, where it responsible for the structural and mechanical support [90]. It is a natural piezoelectric material with piezoelectric coefficient ranges from 0.2 to 2.0 pC/N [79]. Additionally, it is suitable as a biomaterial in tissue engineering due to its biocompatibility, good cell binding properties, hydrophilicity, low antigenicity, absorbability in the body etc. [17]. The researchers had been reported the application of collagen scaffold in bone healing [91]. Similarly the collagen-hydroxyapatite piezoelectric composite scaffold has been proved as a suitable structure for cellular growth and bone healing [92]. Also the collagen-calcium phosphate composite scaffolds are reported for cartilage tissue engineering. Studies with collagen-calcium phosphate composite scaffolds are demonstrated the average filling ratio of the defect area with the newly formed cartilage tissue at week eight and twenty is about 81% and 96%, respectively [80]. However, it has certain limitations like low mechanical stiffness, rapid degradation and toxicity by addition of crosslinking agents.

#### Chitin

Chitin is a natural polysaccharide and is the structural component of the cuticles of crustaceans, insects and mollusks. It has a piezoelectric structure with piezoelectric coefficient ranges from 0.2–1.5 pC/N depending upon its source [79]. Chitosan is a polymer which is obtained by the deacetylation of chitin, has a number of biomedical applications such as wound healing and carriers for controlled drug delivery etc. [93]. By making it composite with other suitable filler components it is suitable for bone and cartilage regeneration. Chitin largely favors for biomedical applications, since it is a hydrophilic material, which promotes cell adhesion, cell proliferation, differentiation and it offers well biocompatibility [17]. But, low mechanical properties and inability to maintain predefined shape, limits its use in tissue engineering particularly for hard tissue applications.

### Piezoceramics

A large number of piezoceramics are available with a very high piezoelectric coefficient, such as lead zirconate titanate (PZT), barium titanate (BT), zinc oxide (ZnO), potassium sodium niobate (KNN), lithium sodium potassium niobate (LNPN) and boron nitride nanotubes (BNNT). The common concern related piezoceramics in tissue engineering is its cytotoxicity. In general, lead contained ceramics have limited application in tissue engineering due to their toxic nature. The PZT possess the very high piezoelectric constant ranges from 200 to 350 pC/N is a highly cytotoxicity [94]. Hence, PZT would not be preferred in tissue engineering application and the lead-free piezo ceramics could be an alternative choice. Other ceramic also have dose dependent toxicity so they are applicable for tissue engineering up to some extent.

#### Barium Titanate

Barium titanate (BT) is highly biocompatible with d_33_ coefficient of 191pC/N [3]. It has been reported that the BT nanoparticles have demonstrated cytocompatibility, even at higher concentrations like 100 μg/ml [95]. The studies have been demonstrated that the PLGA matrix reinforced with BT nanoparticles supports the cell attachment and proliferation of osteoblast and osteocytes [96]. Also, TiO_2_ powders have the ability to improve the osteoconductivity hence have improved efficacy to promote osteoblast adhesion [97]. Significantly, it has been reported that the piezoelectric property of BT has a positive influence on the cellular proliferation [98]. Furthermore, the incorporation of barium titanate nanoparticles into the polymeric matrix would improve the mechanical properties of the composite scaffold structure [99]. Hence, it is quite evident that the piezoelectric BT has an ability to promote the cellular activities in tissue engineering applications.

#### Zinc oxide

Zinc has a critical role in cell proliferation and differentiation in the biological system by modulating the activity of different enzymes including transcription factors, metalloproteinase and polymerases [100]. Piezoelectric zinc oxide has not shown any toxic effects in micrometer and larger size ranges [101], but it has been demonstrated toxicity in nano size due to the production of reactive oxygen species [102]. Significant results has reported on zinc oxide nanoparticles dispersed in the polymeric scaffold along with hypoxia have shown ability to synthesis cartilage [103]**.** According to Material Safe Data Sheet (MSDS) databases LD50 of acute oral ZnO is 7950 mg/kg for mice shows no significant toxicity [104]. Moreover, the cytotoxicity of the nanoparticles can be reduced by chemical and physical modification for medical application [102].

#### Potassium sodium Niobate (KNN) and lithium sodium potassium Niobate (LKNN)

KNN and LKNN are lead free piezoelectric ceramic materials with piezoelectric coefficient 63 pC/N and 98 pC/N, respectively [105]. Addition of lithium (Li) has largely enhanced piezoelectric properties, while it would increase the cytotoxicity due to the release of Li ions when it is exposed to the bioenvironment [65]. The electric charge of the ferroelectric lithium niobate crystals enhances the proliferation and osteoblastic activity to rapid bone regeneration [106]. It has been reported that the utilization of piezoelectric property of KNN in drug delivery devices and also it is applicable for bone, cartilage, skin and nerve repair and regeneration [107].

#### Boron nitride

**Fig. 3:**
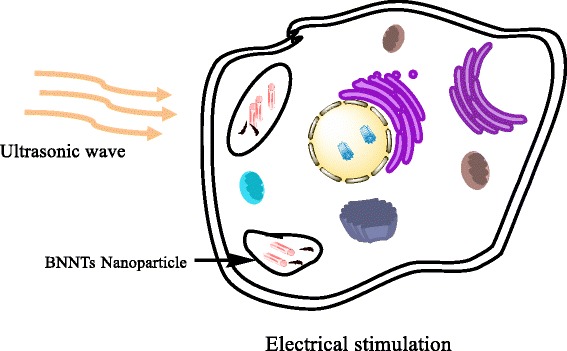
Electrical stimulation to cells by internalized BNNT nanoparticle as a result of external ultrasound irradiation. The direct piezoelectric effect applied on BNNTs and ultrasonic wave as mechanical stress to convert into electrical stimuli for enhanced cell differentiation

Boron nitride nanotube has superior piezoelectric property than that of piezoelectric polymers [108]. Researchers are exploited BNNTs as nano vectors to carry electrical /mechanical stimuli on demand within a cellular system. After BNNT internalization, the electrical stimulation has conveyed to tissue or/ cell culture using a wireless mechanical source (i.e., ultrasound) (Fig. 3). Its cytocompatibility can be improved by improving its dispersibility in the solvents. It is reported that its dispersibility can be improved by non-covalent polymeric wrapping or by using non-toxic surfactants, which has been increased its potential for biomedical application [109]. The proper functionalization of BNNT with glycol-chitosan or the addition of surfactant poly-L-lysine (PLL) or polyethyleneimine (PEI) results in the formation of BNNT dispersion and improves the cytocompatibility of BNNT [110]. The studies have been demonstrated that biodegradable polymeric scaffold reinforced with BNNT has a positive influence on osteoblast proliferation and differentiation [111, 112]. The studies report that the BNNT has a negative influence on the chondrocytes, fibroblast and smooth muscle cells. It decreases the adhesion of chondrocytes, fibroblasts and smooth muscle cells while it can increase the adhesion of osteoblast cells [110]. Moreover, it has excellent mechanical properties and highly crucial for orthopedic applications [113]. Hence BNNT is an excellent material for bone tissue engineering.

### Future prospective

Piezoelectric collagen fibers are present in cartilage and bone, but the function of piezoelectricity is not yet fully investigated. The piezoelectric material can act as a mechanoelectrical transducer. The electroactive scaffolds can generate the electric field in response to minute mechanical vibrations. Also the scaffold of piezoelectric material can be tuned the effective electric field characteristics of the natural ECM observed during development, regeneration or repair of the tissues. The scaffold can directly influence the osteoblast or chondroblast cells and can promote its adhesion and proliferation, further the production of ECM and thereby repair of damaged sites. Moreover it can stimulate the mesenchymal stem cells directly and further its differentiation into chondroblasts or osteoblasts. Therefore, the smart piezoelectric biomaterials require strong attention towards tissue engineering, particularly bone, cartilage and nerve regeneration. These materials will offer natural physiological conditions like ECM to regulate the signaling pathways to stimulate the regeneration mechanism. Significantly, the piezoelectric scaffolds can enhance the cell functionality without the addition of growth factors and drug molecules. The stimulating factors implanted treatments are highly expensive, highly instable (extra and random growth of tissue), complicated selection criteria (lack of dose optimization criteria) and dose related complications. Even more, the stimulating factors implanted scaffolds, further compacted the treatment procedure. Therefore, the smart piezoelectric material based scaffolds can be better alternative to aforementioned conventional therapies. The smart scaffold utilizes the functional loads as stimulating factor to regenerate the tissue by effect. The tissue regeneration can be regulated by natural feedback system to maintain the integration of the system. Hence, the class of piezoelectric materials has huge research and market scope for advanced tissue engineering therapies.

## Conclusion

The present review provides the brief insight about the importance of the alternative technologies like smart materials in regenerative medicine. The detailed information about various piezoelectric materials for bone and cartilage tissue engineering has been presented in the report. Numerous piezoelectric materials are available and proved its effectiveness in the field of sensors; actuators etc. while the exploration of their biomedical applications are exponentially increased in last decade. Piezoelectric polymers/ biopolymers like, PHBV, PLLA, PVDF, collagen and cellulose etc. have been discussed in detail in terms of applications and their physical properties. Piezoceramics have been debated for their applications for hard tissue regeneration with various forms. Hence, the piezoelectric smart materials are best possible futuristic materials for regenerative medicine.

## Abbreviations

BMP: Bone Morphogenetic Protein
BNNT: Boron nitride nanotubes
BT: Barium titanate
ECM: Extracellular matrix
IL-1: Interleukin-1
IL-4: Interleukin-4
KNN: potassium sodium niobate
Li: Lithium
LKNN: Lithium sodium potassium niobate
LNPN: Lithium sodium potassium niobate
NF-AT: Nuclear Factor of Activated Cells
PEI: Polyethylene imine
PHBV: Poly(3-hydroxybutyrate-co-3-hydroxyvalerate)
PKC: Protein kinase
PLL: Poly-L-lysine
PLLA: Poly-L-Lactic acid
PVDF: Poly(vinylidene fluoride)
PZT: Lead zirconate titanate
TGF β: Transforming Growth Factorβ
TrFE: Trifluoroethylene
ZnO: Zinc oxide

## References

[1] Lee SJ, Yoo JJ, Atala A. Biomaterials and tissue engineering. In: Clinical Regenerative Medicine in Urology. Singapore: Springer; 2018. p. 17–51.

[2] Mason WP (1981). Piezoelectricity, its history and applications. The Journal of the Acoustical Society of America.

[3] Mindlin R (1972). Elasticity, piezoelectricity and crystal lattice dynamics. J Elast.

[4] Mould R (2007). Pierre Curie, 1859–1906. Curr Oncol.

[5] Arinzeh T, Collins G, Lee Y-S (2016). System and method for a piezoelectric scaffold for nerve growth and repair. Google Patents.

[6] Minary-Jolandan M (2009). Yu M-F: **nanoscale characterization of isolated individual type I collagen fibrils: polarization and piezoelectricity**. Nanotechnology.

[7] Arinzeh TL, Weber N, Jaffe M (2016). Electrospun electroactive polymers for regenerative medicine applications. Google Patents.

[8] Shastri VR, Schmidt CE, Langer RS, Vacanti JP (2000). Neuronal stimulation using electrically conducting polymers. Google patents.

[9] Bassett CAL (1967). Biologic significance of piezoelectricity. Calcif Tissue Int.

[10] Halperin C, Mutchnik S, Agronin A, Molotskii M, Urenski P, Salai M, Rosenman G (2004). Piezoelectric effect in human bones studied in nanometer scale. Nano Lett.

[11] Miara B, Rohan E, Zidi M, Labat B (2005). Piezomaterials for bone regeneration design—homogenization approach. Journal of the Mechanics and Physics of Solids.

[12] Huey DJ (2012). Hu JC, Athanasiou KA: **unlike bone, cartilage regeneration remains elusive**. Science.

[13] Tuzlakoglu K, Bolgen N, Salgado A, Gomes ME, Piskin E, Reis R (2005). Nano-and micro-fiber combined scaffolds: a new architecture for bone tissue engineering. J Mater Sci Mater Med.

[14] Grodzinsky AJ, Levenston ME, Jin M, Frank EH (2000). Cartilage tissue remodeling in response to mechanical forces. Annu Rev Biomed Eng.

[15] Poole AR, Kojima T, Yasuda T, Mwale F, Kobayashi M, Laverty S (2001). Composition and structure of articular cartilage: a template for tissue repair. Clin Orthop Relat Res.

[16] Huber M, Trattnig S, Lintner F (2000). Anatomy, biochemistry, and physiology of articular cartilage. Investig Radiol.

[17] Puppi D, Chiellini F, Piras A, Chiellini E (2010). Polymeric materials for bone and cartilage repair. Prog Polym Sci.

[18] Eyre D (2001). Articular cartilage and changes in arthritis: collagen of articular cartilage. Arthritis Research & Therapy.

[19] Muir H, Bullough P, Maroudas A (1970). The distribution of collagen in human articular cartilage with some of its physiological implications. Bone & Joint Journal.

[20] Reddi AH (2000). Morphogenesis and tissue engineering of bone and cartilage: inductive signals, stem cells, and biomimetic biomaterials. Tissue Eng.

[21] Lane NE (2007). Metabolic bone disease. Curr Opin Rheumatol.

[22] Mankin HJ (1974). The reaction of articular cartilage to injury and osteoarthritis. N Engl J Med.

[23] Riggs BL, Hartmann LC (2003). Selective estrogen-receptor modulators—mechanisms of action and application to clinical practice. N Engl J Med.

[24] Giusti A, Hamdy NA, Papapoulos SE (2010). Atypical fractures of the femur and bisphosphonate therapy: a systematic review of case/case series studies. Bone.

[25] Allen HL, Wase A, Bear W (1980). Indomethacin and aspirin: effect of nonsteroidal anti-inflammatory agents on the rate of fracture repair in the rat. Acta Orthop Scand.

[26] Rodan GA, Martin TJ (2000). Therapeutic approaches to bone diseases. Science.

[27] Fishman JA, Greenwald MA, Grossi PA (2012). Transmission of infection with human allografts: essential considerations in donor screening. Clin Infect Dis.

[28] Cypher TJ, Grossman JP (1996). Biological principles of bone graft healing. The Journal of foot and ankle surgery.

[29] Jackson DW, Windler GE, Simon TM (1990). Intraarticular reaction associated with the use of freeze-dried, ethylene oxide-sterilized bone-patella tendon-bone allografts in the reconstruction of the anterior cruciate ligament. Am J Sports Med.

[30] Steadman JR, Briggs KK, Rodrigo JJ, Kocher MS, Gill TJ, Rodkey WG (2003). Outcomes of microfracture for traumatic chondral defects of the knee: average 11-year follow-up. Arthroscopy: The Journal of Arthroscopic & Related Surgery.

[31] Nehrer S, Minas T (2000). Treatment of articular cartilage defects. Investig Radiol.

[32] Grande DA, Pitman MI, Peterson L, Menche D, Klein M (1989). The repair of experimentally produced defects in rabbit articular cartilage by autologous chondrocyte transplantation. J Orthop Res.

[33] Nerem RM, Sambanis A (1995). Tissue engineering: from biology to biological substitutes. Tissue Eng.

[34] Wu Q, Yang B, Hu K, Cao C, Man Y, Wang P (2017). Deriving osteogenic cells from induced pluripotent stem cells for bone tissue engineering. Tissue Eng B Rev.

[35] Wan C, He Q, Li G (2006). Allogenic peripheral blood derived mesenchymal stem cells (MSCs) enhance bone regeneration in rabbit ulna critical-sized bone defect model. J Orthop Res.

[36] Nakashima M, Nagasawa H, Yamada Y, Reddi AH (1994). Regulatory role of transforming growth factor-β, bone morphogenetic protein-2, and protein-4 on gene expression of extracellular matrix proteins and differentiation of dental pulp cells. Dev Biol.

[37] Sakaguchi Y, Sekiya I, Yagishita K, Muneta T (2005). Comparison of human stem cells derived from various mesenchymal tissues: superiority of synovium as a cell source. Arthritis & Rheumatology.

[38] More N, Kapusetti G (2017). Piezoelectric material–a promising approach for bone and cartilage regeneration. Med Hypotheses.

[39] van Beuningen HM, Glansbeek HL, van der Kraan PM, van den Berg WB (1998). Differential effects of local application of BMP-2 or TGF-β1 on both articular cartilage composition and osteophyte formation. Osteoarthr Cartil.

[40] Koga H, Engebretsen L, Brinchmann JE, Muneta T, Sekiya I (2009). Mesenchymal stem cell-based therapy for cartilage repair: a review. Knee Surg Sports Traumatol Arthrosc.

[41] Cancedda R, Dozin B, Giannoni P, Quarto R (2003). Tissue engineering and cell therapy of cartilage and bone. Matrix Biol.

[42] Amini AR, Laurencin CT, Nukavarapu SP (2012). Bone tissue engineering: recent advances and challenges. Crit Rev™ Biomed Eng.

[43] Ziegler J, Mayr-Wohlfart U, Kessler S, Breitig D, Günther KP (2002). Adsorption and release properties of growth factors from biodegradable implants. J Biomed Mater Res A.

[44] Mano JF. Smart polymers: applications in biotechnology and biomedicine. In: USA: Wiley Online library; 2009. p. 622.

[45] Reis R, Mano J, Del Campo A. Smart instructive polymer substrates for tissue engineering. In: Smart polymers and their applications. Cambridge: Elsevier; 2014. p. 301–26.

[46] Li S, Tiwari A, Prabaharan M, Aryal S. Smart polymer materials for biomedical applications. New York: Nova Science Publishers, Inc; 2011.

[47] Roy D, Cambre JN, Sumerlin BS (2010). Future perspectives and recent advances in stimuli-responsive materials. Prog Polym Sci.

[48] Del as Heras Alarcón C, Pennadam S, Alexander C (2005). Stimuli responsive polymers for biomedical applications. Chem Soc Rev.

[49] Jeong B, Gutowska A (2002). Lessons from nature: stimuli-responsive polymers and their biomedical applications. Trends Biotechnol.

[50] Ahn AC, Grodzinsky AJ (2009). Relevance of collagen piezoelectricity to “Wolff's law”: a critical review. Med Eng Phys.

[51] Xu J, Wang W, Clark C, Brighton C (2009). Signal transduction in electrically stimulated articular chondrocytes involves translocation of extracellular calcium through voltage-gated channels. Osteoarthr Cartil.

[52] Ballock RT, Heydemann A, Wakefield LM, Flanders KC, Roberts AB, Sporn MB (1993). TGF-β1 prevents hypertrophy of epiphyseal chondrocytes: regulation of gene expression for cartilage matrix proteins and metalloproteases. Dev Biol.

[53] Zhuang H, Wang W, Seldes RM, Tahernia AD, Fan H, Brighton CT (1997). Electrical stimulation induces the level of TGF-β1 mRNA in osteoblastic cells by a mechanism involving calcium/calmodulin pathway. Biochem Biophys Res Commun.

[54] Riddle RC, Donahue HJ (2009). From streaming-potentials to shear stress: 25 years of bone cell mechanotransduction. J Orthop Res.

[55] Fukada E, Yasuda I (1964). Piezoelectric effects in collagen. Jpn J Appl Phys.

[56] Spadaro JA (1997). Mechanical and electrical interactions in bone remodeling. Bioelectromagnetics.

[57] Huang C, Ogawa R (2010). Mechanotransduction in bone repair and regeneration. FASEB J.

[58] Lee H-S, Millward-Sadler S, Wright M, Nuki G, Al-Jamal R, Salter D (2002). Activation of integrin—RACK1/PKCα signalling in human articular chondrocyte mechanotransduction. Osteoarthr Cartil.

[59] Litzenberger JB, Kim J-B, Tummala P, Jacobs CR (2010). β1 integrins mediate mechanosensitive signaling pathways in osteocytes. Calcif Tissue Int.

[60] Kanno T, Takahashi T, Tsujisawa T, Ariyoshi W, Nishihara T (2007). Mechanical stress-mediated Runx2 activation is dependent on Ras/ERK1/2 MAPK signaling in osteoblasts. J Cell Biochem.

[61] Li C, Weng G. Antiplane crack problem in functionally graded piezoelectric materials. TRANSACTIONS-AMERICAN SOCIETY OF MECHANICAL ENGINEERS. J Appl Mech. 2002;69(4):481–8.

[62] Berlincourt D, Cmolik C, Jaffe H (1960). Piezoelectric properties of polycrystalline lead titanate zirconate compositions. Proc IRE.

[63] Fousek J, Cross L, Litvin D (1999). Possible piezoelectric composites based on the flexoelectric effect. Mater Lett.

[64] Setter N, Damjanovic D, Eng L, Fox G, Gevorgian S, Hong S, Kingon A, Kohlstedt H, Park N, Stephenson G (2006). Ferroelectric thin films: review of materials, properties, and applications. J Appl Phys.

[65] Rajabi AH, Jaffe M, Arinzeh TL (2015). Piezoelectric materials for tissue regeneration: a review. Acta Biomater.

[66] Gimenes R, Zaghete MA, Bertolini M, Varela JA, Coelho LO, Silva NF Jr. Composites PVDF-TrFE/BT used as bioactive membranes for enhancing bone regeneration. In: Smart structures and materials. California: International Society for Optics and Photonics; 2004. p. 539–47.

[67] Martins P, Ribeiro S, Ribeiro C, Sencadas V, Gomes A, Gama F, Lanceros-Méndez S (2013). Effect of poling state and morphology of piezoelectric poly (vinylidene fluoride) membranes for skeletal muscle tissue engineering. RSC Adv.

[68] Ribeiro C, Pärssinen J, Sencadas V, Correia V, Miettinen S, Hytönen VP, Lanceros-Méndez S (2015). Dynamic piezoelectric stimulation enhances osteogenic differentiation of human adipose stem cells. J Biomed Mater Res A.

[69] Reis J, Frias C, Canto e Castro C, Botelho ML, Marques AT, JAO S, Capela e Silva F, Potes J. A new piezoelectric actuator induces bone formation in vivo: a preliminary study. Biomed Res Int. 2012;2012:613403. 10.1155/2012/613403 PMC3369535 22701304

[70] Lee Y-S, Livingston Arinzeh T (2011). Electrospun nanofibrous materials for neural tissue engineering. Polymers.

[71] Ross G, Watts J, Hill M, Morrissey P (2000). Surface modification of poly (vinylidene fluoride) by alkaline treatment1. The degradation mechanism. Polymer.

[72] Neuss S, Apel C, Buttler P, Denecke B, Dhanasingh A, Ding X, Grafahrend D, Groger A, Hemmrich K, Herr A (2008). Assessment of stem cell/biomaterial combinations for stem cell-based tissue engineering. Biomaterials.

[73] Fukada E (1998). New piezoelectric polymers. Jpn J Appl Phys.

[74] Weber N, Lee Y-S, Shanmugasundaram S, Jaffe M, Arinzeh TL (2010). Characterization and in vitro cytocompatibility of piezoelectric electrospun scaffolds. Acta Biomater.

[75] Valentini RF (1998). Negatively charged polymeric electret implant. Google patents.

[76] Pereira JD, Camargo RC, José Filho C, Alves N, Rodriguez-Perez MA, Constantino CJ (2014). Biomaterials from blends of fluoropolymers and corn starch—implant and structural aspects. Mater Sci Eng C.

[77] Esmaeili M, Baei MS (2011). Fabrication of biodegradable polymer nanocomposite from copolymer synthesized by *C. necator* for bone tissue engineering.

[78] Ke S, Huang H, Ren L, Wang Y (2009). Nearly constant dielectric loss behavior in poly (3-hydroxybutyrate-co-3-hydroxyvalerate) biodegradable polyester. AIP.

[79] Fukada E (2000). History and recent progress in piezoelectric polymers. IEEE Trans Ultrason Ferroelectr Freq Control.

[80] Köse GT, Korkusuz F, Özkul A, Soysal Y, Özdemir T, Yildiz C, Hasirci V (2005). Tissue engineered cartilage on collagen and PHBV matrices. Biomaterials.

[81] Numata K, Abe H, Doi Y (2008). Enzymatic processes for biodegradation of poly (hydroxyalkanoate) s crystals. Can J Chem.

[82] Newman B, Chen P, Pae K, Scheinbeim J (1980). Piezoelectricity in nylon 11. J Appl Phys.

[83] Takahashi Y, Iijima M, Fukada E (1989). Pyroelectricity in poled thin films of aromatic polyurea prepared by vapor deposition polymerization. Jpn J Appl Phys.

[84] Wang H, Li Y, Zuo Y, Li J, Ma S, Cheng L (2007). Biocompatibility and osteogenesis of biomimetic nano-hydroxyapatite/polyamide composite scaffolds for bone tissue engineering. Biomaterials.

[85] Naughton GK, Willoughby J (1998). Method for repairing cartilage. Google Patents.

[86] Fukada E (1995). Piezoelectricity of biopolymers. Biorheology.

[87] Di Martino A, Sittinger M, Risbud MV (2005). Chitosan: a versatile biopolymer for orthopaedic tissue-engineering. Biomaterials.

[88] Kim J, Yun S, Ounaies Z (2006). Discovery of cellulose as a smart material. Macromolecules.

[89] Zaborowska M, Bodin A, Bäckdahl H, Popp J, Goldstein A, Gatenholm P (2010). Microporous bacterial cellulose as a potential scaffold for bone regeneration. Acta Biomater.

[90] Ferreira AM, Gentile P, Chiono V, Ciardelli G (2012). Collagen for bone tissue regeneration. Acta Biomater.

[91] Rocha LB, Goissis G, Rossi MA (2002). Biocompatibility of anionic collagen matrix as scaffold for bone healing. Biomaterials.

[92] Silva C, Thomazini D, Pinheiro A, Aranha N, Figueiro S, Goes J, Sombra A (2001). Collagen–hydroxyapatite films: piezoelectric properties. Mater Sci Eng B.

[93] Silva C, Lima C, Pinheiro A, Góes J, Figueiro S, Sombra A (2001). On the piezoelectricity of collagen–chitosan films. Phys Chem Chem Phys.

[94] Savakus H, Klicker K, Newnham R (1981). PZT-epoxy piezoelectric transducers: a simplified fabrication procedure. Mater Res Bull.

[95] Ciofani G, Ricotti L, Canale C, D’Alessandro D, Berrettini S, Mazzolai B, Mattoli V (2013). Effects of barium titanate nanoparticles on proliferation and differentiation of rat mesenchymal stem cells. Colloids Surf B: Biointerfaces.

[96] Ciofani G, Ricotti L, Mattoli V (2011). Preparation, characterization and in vitro testing of poly (lactic-co-glycolic) acid/barium titanate nanoparticle composites for enhanced cellular proliferation. Biomed Microdevices.

[97] Liu H, Slamovich EB, Webster TJ (2006). Increased osteoblast functions among nanophase titania/poly (lactide-co-glycolide) composites of the highest nanometer surface roughness. J Biomed Mater Res A.

[98] Baxter FR, Bowen CR, Turner IG, Dent AC (2010). Electrically active bioceramics: a review of interfacial responses. Ann Biomed Eng.

[99] Ivanova O, Williams C, Campbell T (2013). Additive manufacturing (AM) and nanotechnology: promises and challenges. Rapid Prototyp J.

[100] Shankar AH, Prasad AS (1998). Zinc and immune function: the biological basis of altered resistance to infection. Am J Clin Nutr.

[101] Fan Z (2005). Lu JG: **zinc oxide nanostructures: synthesis and properties**. J Nanosci Nanotechnol.

[102] Rasmussen JW, Martinez E, Louka P, Wingett DG (2010). Zinc oxide nanoparticles for selective destruction of tumor cells and potential for drug delivery applications. Expert opinion on drug delivery.

[103] Mirza EH, Pan-Pan C, Ibrahim W, Bin WMA, Djordjevic I, Pingguan-Murphy B (2015). Chondroprotective effect of zinc oxide nanoparticles in conjunction with hypoxia on bovine cartilage-matrix synthesis. J Biomed Mater Res A.

[104] Wang B, Feng W, Wang M, Wang T, Gu Y, Zhu M, Ouyang H, Shi J, Zhang F, Zhao Y (2008). Acute toxicological impact of nano-and submicro-scaled zinc oxide powder on healthy adult mice. J Nanopart Res.

[105] Yu S-W, Kuo S-T, Tuan W-H, Tsai Y-Y, Wang S-F (2012). Cytotoxicity and degradation behavior of potassium sodium niobate piezoelectric ceramics. Ceram Int.

[106] Carville NC, Collins L, Manzo M, Gallo K, Lukasz BI, McKayed KK, Simpson JC, Rodriguez BJ (2015). Biocompatibility of ferroelectric lithium niobate and the influence of polarization charge on osteoblast proliferation and function. J Biomed Mater Res A.

[107] Atanasoska L, Radhakrishnan R, Schewe S (2014). Medical devices employing piezoelectric materials for delivery of therapeutic agents. Google patents.

[108] Ciofani G, Raffa V, Menciassi A, Cuschieri A (2009). Boron nitride nanotubes: an innovative tool for nanomedicine. Nano Today.

[109] Ciofani G, Raffa V, Menciassi A, Dario P (2008). Preparation of boron nitride nanotubes aqueous dispersions for biological applications. J Nanosci Nanotechnol.

[110] Ciofani G, Danti S, Genchi GG, Mazzolai B, Mattoli V (2013). Boron nitride nanotubes: biocompatibility and potential spill-over in nanomedicine. Small.

[111] Ciofani G, Danti S, D’Alessandro D, Ricotti L, Moscato S, Bertoni G, Falqui A, Berrettini S, Petrini M, Mattoli V (2010). Enhancement of neurite outgrowth in neuronal-like cells following boron nitride nanotube-mediated stimulation. ACS Nano.

[112] Lahiri D, Rouzaud F, Richard T, Keshri AK, Bakshi SR, Kos L, Agarwal A (2010). Boron nitride nanotube reinforced polylactide–polycaprolactone copolymer composite: mechanical properties and cytocompatibility with osteoblasts and macrophages in vitro. Acta Biomater.

[113] Nakhmanson SM, Calzolari A, Meunier V, Bernholc J, Nardelli MB (2003). Spontaneous polarization and piezoelectricity in boron nitride nanotubes. Phys Rev B.

